# Effectiveness of visceral fascial therapy targeting visceral dysfunctions outcome: systematic review of randomized controlled trials

**DOI:** 10.1186/s12906-023-04099-1

**Published:** 2023-07-31

**Authors:** Fabiana C. da Silva, Leonardo S. Vieira, Lucas V. Santos, Nathaly Gaudreault, Ronaldo H. Cruvinel-Júnior, Gilmar M. Santos

**Affiliations:** 1 Cirklo Health Education, Porto Alegre, Rio Grande do Sul Brazil; 2 Abfáscias, Belo Horizonte, Minas Gerais Brazil; 3 Brazilian College of Osteopathy, Sorocaba, São Paulo, Brazil; 4 Faculty of medicine and health sciences, University of Sherbrooke, Sherbrooke, Canada; 5 Department of Physical Therapy, Speech, and Occupational Therapy, School of Medicine, University of São Paulo, São Paulo, Brazil; 6 Physical Therapy Graduate Program, Health, and Sports Sciences Center, Santa Catarina State University, Florianopolis, Santa Catarina Brazil

**Keywords:** Fascial therapy, Visceral manipulation therapy, Visceral disorders, Pain

## Abstract

**Background:**

Fascial Therapy is an ancient and widespread practice throughout the world. These approaches are very common in osteopathic practice and taught in workshops for professionals from different areas of health care, including Physiotherapy. This type of treatment is quite specialized and centered on the therapist. However, there is a lack of high-quality and low-risk bias studies that justify the use of this practice. Despite this, there is little scientific evidence about the effectiveness of Fascial Therapy to treat some visceral disorders. The purpose of this study was to critically appraise the scientific literature concerning the clinical efficacy of techniques used in Fascial Therapy targeting the visceral system.

**Methods:**

This systematic review included randomized controlled trials in any language or date of publication. All primary outcomes reported were included. The methodological quality and statistical reporting of each eligible trial were evaluated using the version 2 of the Cochrane risk-of-bias tool for randomized trials (RoB 2). This systematic review provided a synthesis of current evidence on the effects of Fascial Therapy in patients with visceral disorders and/or pain. A total of 11 studies were included, with five of them covering gastrointestinal dysfunction, two covering cardiorespiratory dysfunction, two covering musculoskeletal dysfunction, and two covering urogenital dysfunction.

**Results:**

Fascial Therapy targeting the visceral system has been shown to be effective in reducing pain over the long term in people with low back pain when combined with standard physical therapy and effective in reducing gastroesophageal reflux symptoms over the short term. Considering the overall bias, six studies were at high risk of bias, two studies had some concerns and only three studies were at low risk of bias. Of the three studies with a low risk of bias, only two showed positive results and were effective in improving the studied outcome.

**Conclusion:**

This systematic review shows that currently, there is poor evidence for the efficacy of the techniques used in Fascial Therapy targeting the visceral system, and this information can help healthcare professionals in decision-making related to the use of Fascial Therapy targeting the visceral system in patients with visceral disorders and/or pain.

**Supplementary Information:**

The online version contains supplementary material available at 10.1186/s12906-023-04099-1.

## Introduction

Chronic musculoskeletal pain represents a significant burden on global health [1]. Approximately one in three Americans suffer from chronic pain [2], with about one-third of this population experiencing chronic pain associated with chronic visceral comorbidities [3, 4]. A longitudinal cohort study, involving 58,458 individuals, revealed a strong correlation between chronic pain and visceral dysfunctions [5]. Chronic pain of visceral origin falls under the category of secondary chronic pain in the IASP 2019 classification, and it is the subcategory with the highest impact and prevalence worldwide [6].

Fascial therapy (FT) is a common therapeutic option used by clinicians in manual therapy. It involves manual techniques applied to the fascial tissue surrounding internal organs and is often employed to relieve visceral dysfunction and pain [7–9]. Despite its wide usage in clinical practice, there is limited scientific evidence regarding its effectiveness in targeting the visceral system.

Data from practice profiles of Australian osteopaths suggest that the use of manual therapy techniques on the viscera is a part of osteopathy practice [10, 11]. However, fascial therapy targeting the visceral system (FTTVS) is not included in the basic physiotherapy graduation training, and many physiotherapists and health practitioners are seeking additional training in this treatment technique through post-graduate workshops. FTTVS involves light or deep manual fascial releases and specific organ mobilizations in the thoracic, subdiaphragmatic, abdominal, and pelvic areas [7].

Several previous studies have demonstrated the effects of FTTVS in the treatment of chronic low back and neck pain in individuals with limited visceral mobility [12, 13]. Vaca et al. (2019) published a scoping review of visceral changes associated with pain and mobility problems in the cervical region [14]. However, Guillaud et al. (2018) reported low diagnostic reliability and clinical efficacy of FTTVS in a published systematic review [15].

In 2021, Lo Basso et al. conducted a randomized controlled trial to assess whether manual treatment relieves Urinary Tract Infection and reduces pain in patients with nonspecific LBP through improvement in kidney mobility. They found that patients who received thrust manipulation and FTTVS had significantly improved mean mobility and LBP scores compared to those who only received FTTVS [16]. These results suggest that FTTVS may have limited efficacy when used alone and could be more effective when combined with other manual therapy techniques.

While a systematic review was published in 2018, there is a notable number of recently conducted randomized controlled trials (RCTs) that warrant an updated review. Additionally, this review is justified by its focus on including RCTs that employ different approaches and techniques, including work on visceral fascia, beyond the scope of osteopathic approaches. The findings from this review can assist clinicians in making evidence-based clinical decisions. Therefore, the purpose of this study is to critically appraise the scientific literature concerning the clinical efficacy of fascial therapy targeting the visceral system.

## Methods

A systematic review was performed according to the Preferred Reporting Items for Systematic Reviews and Meta-Analyses (PRISMA) guidelines [17] and prospectively registered in the PROSPERO database for systematic reviews in 2022 (CRD42022345614). Initially, the population of interest, interventions, and outcomes (PICOs) were defined, and the research question was formulated accordingly.

### Population

The population of interest for this systematic review was people aged 16 or older with any pathology/or condition that affects or is related to the visceral system (any condition and duration).

### Interventions

Interventions were selected by the authors based on the literature and their clinical experience and included any type of FTTVS (Osteopathic Manipulative Treatment, Osteopathic Manipulation, Visceral Manipulation, Visceral Osteopathic Manipulation, Visceral Manual Therapy, Visceral Osteopathic Manual Therapy Visceral Osteopathy, Visceral Osteopathic Manipulative Treatment and Visceral Osteopathic treatment).

### Outcomes

For this study were considered any outcome that is related to the visceral system and described all functional measures, tests, or scales that the studies performed to evaluate these outcomes.

### Inclusion and exclusion criteria

RCTs are the gold standard for evaluating the effectiveness of a treatment, as they involve randomly allocating participants to the treatment or control group, which ensures any observed differences can be attributed to the treatment. Systematic reviews that only include RCTs aim to provide a comprehensive summary of high-quality evidence, increasing the review’s internal validity, accuracy, and precision of treatment effects. RCTs are less likely to be affected by bias and confounding compared to other study designs, ensuring the evidence is robust and less likely to be due to chance [18]. Therefore, all RCTs including the interventions of interest were included. We excluded non-randomized clinical trials, pilot studies, cross-sectional studies, case series, case reports, studies involving animal models, technical notes, and feasibility, tolerance, or safety studies. No restriction was made concerning the year of publication of the studies.

### Search strategy

In September 2022, the following databases were searched: PubMed (National Library of Medicine), ScienceDirect, PEDro (Physiotherapy Evidence Database), BVS Bireme, Scielo, CENTRAL, Osteopathic Research Web, Journal of American Osteopathic Association (JAOA) website, OSTMED.D, and CINAHL.

Our search strategy was composed of the following terms that were identified a priori: “osteopathic manipulation” OR “osteopathic manipulative treatment” OR “osteopathic manipulative treatments” OR “visceral manipulation” OR “visceral manual therapy” OR “visceral osteopathy” OR “osteopathic visceral manipulation AND “randomized clinical trials” OR “RCT” (Supplementary file 1).

We also checked the references of all included publications to identify additional publications to be included for assessment.

### Eligibility assessment

The titles and abstracts were analyzed by two independent reviewers blinded to each other’s findings. In cases of divergence, a third researcher (L.S.V) was asked to perform the analysis. When the title and abstract did not contain enough information for the decision regarding eligibility, the full text was read by the two researchers. No restrictions were imposed regarding the minimum sample size. Articles not reporting original research data (books, theoretical articles, and secondary reviews), systematic reviews, and studies not performed with the other evaluation or intervention, were excluded. All studies identified were analyzed after the duplicates were removed. We used the online application Rayyan QCRI for eligibility assessment [19].

### Assessment of included publications

Two reviewers (F.C.S. and L.V.S.) independently assessed included publications for methodological quality, using a revised tool to assess the risk of bias in randomized trials (RoB 2) [20]. RoB 2 is structured into five bias domains: Bias arising from the randomization process, bias due to deviations from intended interventions, bias due to missing outcome data, bias in the measurement of the outcome, and bias in the selection of the reported result. The overall risk of bias generally corresponds to the worst risk of bias in any of the domains. However, if a study is judged to have “some concerns” about the risk of bias for multiple domains, it might be judged as at high risk of bias overall. A judgment as to the possible risk of bias in each of the five domains was made from the extracted information, rated as ‘high risk’, some concerns, or ‘low risk’.

Reviewers resolved disagreement regarding the risk of bias by discussion until a consensus was reached. Data were extracted from each included publication and summarized in evidence tables. These data included participant and study characteristics, characteristics of the intervention and control conditions, and primary and secondary outcomes. One of the reviewers extracted the data, and the other reviewer checked the data for content. All authors thoroughly discussed the evidence tables. In addition to the general assessment of the risk of bias, the results of the studies were analyzed and interpreted.

## Results

**Fig. 1:**
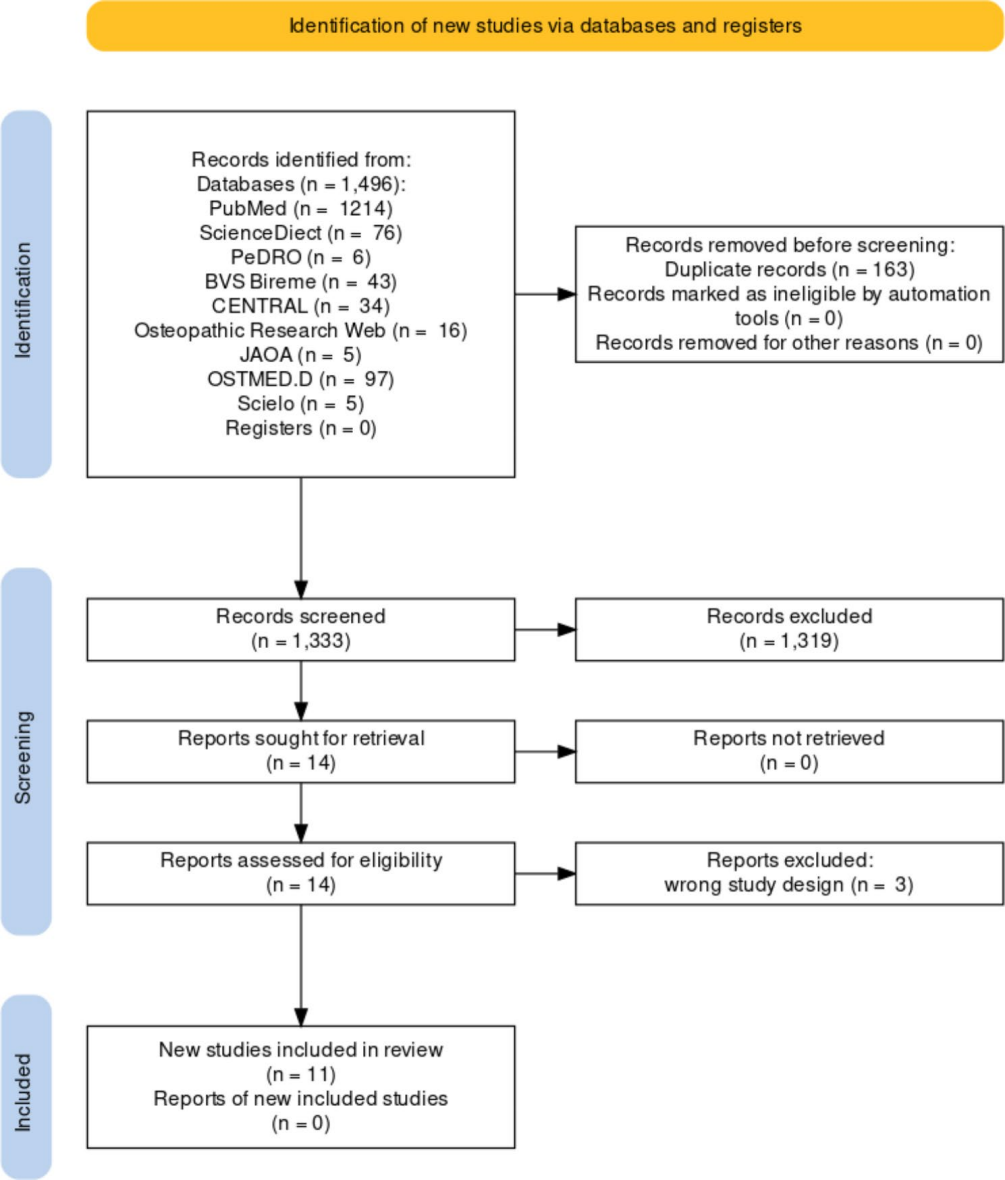
Flowchart of the search process

A total of 1496 articles were retrieved from the electronic databases, based on the keywords. A total of 163 articles were identified as duplicates and were removed. After the full-text analysis, 11 studies were included in this review (Fig. 1).

### Basic characteristics of included studies

The characteristics of the included studies are summarized in Table 1. The studies were published between 2013 and August 2022. The studies included women and men aged between 18 and 80 years. Almost all studies were parallel RCTs, being only one of them a crossover design [21]. The total sample size of the included studies was 490, of which 261 were from the experimental group and 229 were from the control group.

**Table 1:** Descriptive characteristics of the included studies

First author/year	Condition	Sample Size	Treatment	Control	Treatment Schedule	Outcomes measures	Results	Risk of Bias
Attali 2013	Irritable Bowel Syndrome	31	Visceral Osteopathic Manipulation: global visceral technique, the local visceral technique according to highly sensitive zones, and sacral technique.	Placebo (same duration and places treated in the experimental intervention without manipulating visceral tissue)	Cross-over. Only one session.	10 cm Visual Analogue Scales: Constipation, Diarrhea, Abdominal Distension, and Abdominal Pain. Rectal Sensitivity. Total and Segmental Colonic Transit Time.	In a global analysis of the cross-over trial, the IG shows significant decreases in self-reported diarrhea, abdominal distension, and abdominal pain without a change of VAS constipation. The between-group analysis was not performed.	High
Stepnik 2020	Respiratory function in healthy individuals	30	3 techniques: Supine thoracic thrust manipulation, Sternal pump, sternal recoil, and Diaphragm stretch in a sitting position	Placebo (soft tissue therapy for the masseter muscle)	Only one session	Spirometry parameters: Forced vital capacity, forced expiratory volume in 1 s and peak expiratory flow	There were no significant differences between the groups. PEF significantly increased in the IG.	High
Thomaz 2017	Heart failure patients	22	Osteopathy manual therapy: six selected osteopathy techniques (cranial, myofascial, and visceral techniques). Each technique was performed for 2 min with a full completed session lasting 15 min.	Subjects in a supine position for 15 min without intervention	Only one session	Doppler: blood pressure, heart rate, and of blood flow in the carotid, brachial and femoral arteries.	There were no statistical differences between groups. No differences were found between pre and post-test in the control or intervention groups	Some concerns
Piche 2014	Irritable Bowel Syndrome associated with Crohn’s disease	38	Standardized osteopathy (Spinal manipulation and visceral osteopathy). Each session was performed for 60 min.	Three visits with an osteopath during which the osteopath offers caring attention and listening without any manipulation.	Three sessions were performed at 15, 30, and 45 days after the last perfusion of anti-tumor necrosis factor-α (TNF-α) (infliximab).	Irritable bowel syndrome symptoms (IBS severity scoring system); Fatigue Impact Scale, the Beck Depression Inventory, and the Hospital Anxiety and Depression Scale.	There were no statistical differences between groups. Compared with the baseline, the severity of IBS-like symptoms were significantly reduced in the IG (At days 30,45 and 60) with a concomitant increase in Qol (At days 30 and 45). Compared with the baseline, the severity of fatigue was significantly reduced in the IG whereas depression and anxiety remained unchanged.	High
Neto 2020	Stroke Survivors	30	Physical therapy plus visceral manipulation (mobilization of the ascending colon, descending colon, sigmoid colon, and sphincters)	Physical therapy and sham mobilization were performed	Five intervention sessions were held over two weeks.	A ten-item intestinal symptoms rating scale was used to measure the intensity of intestinal Symptoms and static balance were evaluated using a force plate.	A statistically significant intra-group difference was found in the IG regarding the intensity of intestinal symptoms, anteroposterior sway, the velocity of anteroposterior sway, and the velocity of mediolateral sway. No statistically significant differences between groups were found regarding any of the variables related to plantar pressure (static balance).	Low
Panagopoulos 2015	Patients with low back pain	64	Standard physiotherapy plus specific visceral manipulation techniques (5–10 min) - light or deep manual fascial releases and specific organ mobilizations in the thoracic, subdiaphragmatic, abdominal and pelvic areas as appropriate.	Standard physiotherapy plus placebo visceral Manipulation (5 min of sham treatment)	All participants were treated one to two times per week for a minimum of one week and a maximum of 12 treatments over 6 weeks. For both groups, initial treatment sessions lasted for approximately 40 min and follow-up sessions lasted approximately 25–30 min.	The pain was measured with the 0–10 Numerical Pain Rating Scale, disability, with the 0–24 Roland- Morris Disability Scale, and function with the Patient-Specific Functional Scale.	The addition of visceral manipulation did not affect the primary outcome of pain at 6 weeks (− 0.12, 95% CI = − 1.45 to 1.21). There were no significant between-group differences for the secondary outcomes of pain at 2 weeks or disability and function at 2, 6, or 52 weeks. The group receiving the addition of visceral manipulation had less pain than the placebo group at 52 weeks (mean 1.57, 95% CI = 0.32 to 2.82).	low
Yosri 2022	Menstrual complaints in women with polycystic ovarian syndrome	30	Visceral manipulation of the pelvic organs and their related structures over eight sessions, along with the low-calorie diet.	The Control group followed a low-calorie diet (standard care)	The interventions lasted for a total of 3 months.	The study’s primary outcome was the severity of menstrual problems evaluated by the Polycystic Ovary Syndrome Health-Related Quality of Life Questionnaire. The secondary outcomes were weight and BMI, measured by a weight–height scale.	There was a statistically significant reduction in weight, and BMI for the diet group and the diet + VM group). For the improvement in the menstrual complaints, a significant increase in the menstruation domain means the score was shown in the diet group and the diet + VM group. On comparing both groups post-study, there was a statistically significant improvement in the severity of menstruation-related problems in favor of the diet + VM group.	High
De Marco 2022	Urinary Incontinence	52	Pelvic Floor Muscle Training and Manual Visceral Therapy - slow and deep mobilizations over the abdominal and pelvic visceral fasciae.	Pelvic Floor Muscle Training and Manual Sham Therapy - gentle techniques were applied to the thoracic spine, scapular waist, and cervical spine.	20 sessions of Pelvic Floor Muscle Training and 5 sessions of Manual Therapy (experimental or sham)	Urinary Incontinence symptoms: ICQ-UI-SF. Vaginal Resting Pressure and Maximum Voluntary Contraction: Manometry.	There were no significant differences between groups for all outcomes.	High
Lagrange 2019	Incidence of nausea, constipation, and quality of life in women operating for breast cancer and during chemotherapy	94	Visceral manipulation, consisting of the chest wall and diaphragm muscle relaxation through manual thoracic compression	Superficial/soft tissue manipulation without acting on the deeper chest wall and abdominal structures	3 sessions.	Incidence of nausea and vomiting. Constipation. Quality of Life: European Organization for Research and Treatment of Cancer (EORTC) QLQ-C30.	There were no significant differences between groups for all outcomes.	Some concerns
Tamer 2017	Chronic Nonspecific Low Back Pain	39	Visceral Osteopathic Manipulation. All techniques implemented in the control group and thorax, lymphatic, and liver pumping techniques, pelvic floor, diaphragm relaxation techniques	Osteopathic Manipulation Technique. Soft-tissue mobilizations, muscle energy techniques, manipulation, and mobilization for lumbar segment techniques. Exercise approaches were implemented, consisting of spinal stabilization, strengthening, and stretching exercise.	10 sessions for five weeks at two sessions per week.	Pain intensity: Visual Analogue Scale. Quality of Life: SF-36. Functionality: Oswestry Function Scale.	There was no significant difference between groups for all outcomes, except for sub-parameters in SF-36.	High
Eguaras 2019	Gastroesophageal Reflux	60	Visceral Osteopathic Manipulation. The deep manual technique is applied over the epigastric region.	Sham Technique. Superficial contact without any pressure over the epigastric region.	2 sessions.	Gastroesophageal reflux symptoms: GerdQ test. Pressure Pain Threshold (PPT): Algometer. Cervical Mobility: Goniometry.	There were significant differences between groups in the gastroesophageal reflux symptoms one week after intervention, PPT in C4, and cervical mobility.	Low

This table presents the name of the first author of the study and the respective year of publication. It also describes the condition/disease presented by the participants of the study, as well as the sample size. Additionally, the table provides a brief description of the treatment received by the participants and what was offered to the control group (e.g., placebo). Furthermore, we list the outcomes evaluated in each study and their main results. Finally, the risk of bias in the studies was presented. **Abbreviations**: Forced Vital Capacity (FVC), Forced Expiratory Volume in 1 s (FEV1), Peak Expiratory Flow (PEF), fourth cervical vertebra (C4), Body Mass Index (BMI), Visceral Manipulation (VM), Intervention Group (IG), Control Group (CG), International Consultation on Incontinence Questionnaire-Urinary Incontinence Short Form (ICQ-UI-SF), 36-item short-form (SF-36).

### Effects of fascial therapies targeting the visceral system

The overall results are shown in Table 1. FTTVS was well tolerated since there were no side effects reported. A brief sensation of fatigue was reported immediately after FTTVS only in one study [22].

#### Gastrointestinal dysfunction

Attali et al. 2013 conducted a cross-over RCT to evaluate the effects of FTTVS in 31 people with Irritable Bowel Syndrome and in a global analysis of the cross-over trial the participants who received FTTVS showed a significant decrease in self-reported diarrhea, abdominal distension, and abdominal pain without change of constipation visual analog scale [21].

Piche et al., in 2014 conducted an RCT to evaluate the effects of standardized FTTVS in 38 people with Irritable Bowel Syndrome associated with Crohn’s disease, and there were no statistical differences between groups regarding the severity of symptoms, fatigue, depression, and anxiety [23].

An RCT was performed by Lagrange (2019) to evaluate the effects of FTTVS on the incidence of nausea, constipation, and quality of life in 94 women operating on breasts, and there were no significant differences between groups for all outcomes [24].

An RCT was performed by Eguaras (2019) to evaluate the effects of FTTVS on 60 patients with gastroesophageal reflux, and there were significant differences between groups in the gastroesophageal reflux symptoms one week after intervention, pressure pain threshold in the cervical region and cervical mobility [25].

An RCT by Neto (2020) aimed to evaluate the effects of physical therapy plus FTTVS compared to placebo on 30 stroke survivors, and there were no statistical differences between groups regarding the intensity of intestinal symptoms and any of the variables related to plantar pressure (static balance) [26].

#### Cardiorespiratory dysfunction

An RCT by Stepnik (2020) aimed to evaluate the effects of FTTVS on Respiratory function in 30 healthy individuals, and there were no significant differences between groups regarding all spirometry parameters evaluated [27].

An RCT was performed by Thomaz (2017) to evaluate the effects of FTTVS including visceral techniques on 22 heart failure patients, and there were no statistical differences between groups regarding all doppler parameters evaluated [22].

#### Musculoskeletal dysfunction

Panagopoulos et al. conducted an RCT to evaluate the effects of standard physiotherapy plus FTTVS on 64 patients with low back pain, and the results showed that the intervention group had less pain than the placebo group at 52 weeks [28].

Tamer et al. conducted an RCT to evaluate the effects of FTTVS on 39 patients with chronic nonspecific low back pain, and there were no significant differences between groups regarding pain intensity, quality of Life, and functionality [29].

#### Urogenital dysfunction

An RCT was performed by Yosri (2022) to evaluate the effects of FTTVS on the pelvic organs, along with the low-calorie diet on 30 women with polycystic ovarian syndrome accompanied by menstrual complaints, and comparing both groups post-study there was a statistically significant improvement in the severity of menstruation-related problems in favor of the intervention group [30].

De Marco et al. conducted an RCT to evaluate the effects of pelvic floor muscle training and FTTVS on 52 women with urinary incontinence, and there were no statistical differences between groups regarding urinary incontinence symptoms, vaginal resting pressure, and maximum voluntary contraction [31].

### Risk of bias

**Fig. 2:**
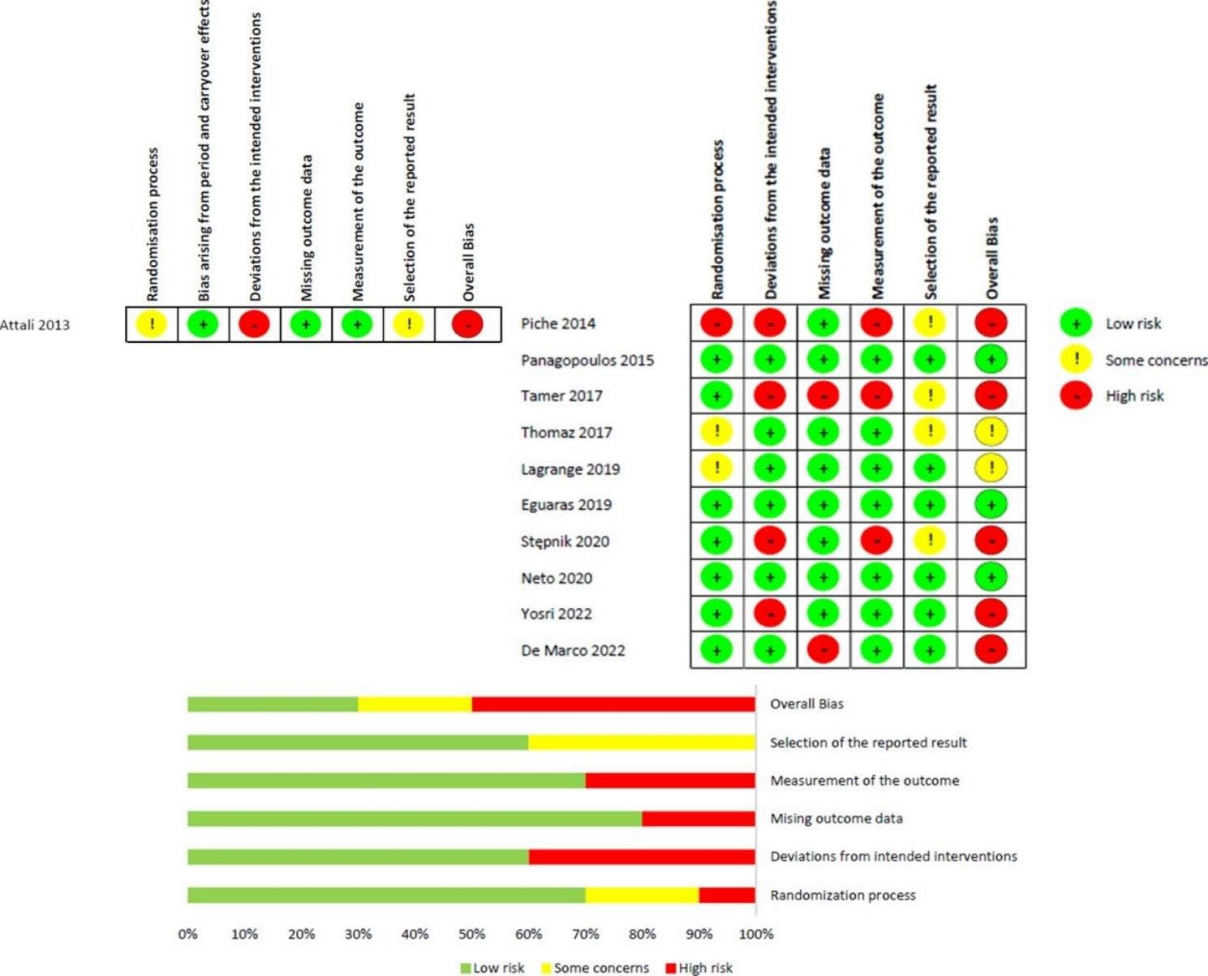
The result of the methodological risk of bias assessment

The risk of bias in the 11 studies was assessed, and a consensus was reached after discussion among two different reviewers (L.V.S and R.H.C.-J). It is important to mention that 10 studies included in this review are parallel randomized controlled clinical trials, and only one study [21] is a crossover randomized controlled clinical trial. Therefore, it was analyzed separately because the RoB 2 scale for risk of bias analysis presents an additional domain for analyzing the risk of bias (bias arising from period and carryover effects). The overall results are shown in Fig. 2.

Four studies were not clear in reporting the participant’s randomization process [21–24], five studies had deviations from the intended interventions [21, 23, 27, 29, 30], two had missing outcome data [29, 31], three had problems in the measurement of the outcome [23, 27, 29], five had some concerns in the selection of the reported result [21–23, 27, 29]. Considering the overall bias six (54.5%) studies were at high risk of bias, two (18.2%) studies had some concerns and only three (27.3%) studies were at low risk of bias.

Additional issues in studies were found, such as no post-test corrections (e.g.: Bonferroni, Tukey, etc.) were implemented to control for inflated alpha values [21, 23–27, 29, 30], the absence of interpretation of the clinical relevance of the results, no effect size calculation and no Confidence Interval 95% reporting [21–24, 26, 27, 29, 30], and no sample size calculation [21, 22, 26, 27, 29].

## Discussion

This review has aimed to identify and critically appraise the scientific studies regarding the clinical efficacy of techniques used in FTTVS. Poor evidence was found for these techniques and only three studies were at low risk of bias. Of the three studies with a low risk of bias, only two [25, 28] showed positive results and were effective in improving the studied outcome. Therefore, the FTTVS has been shown to be effective in reducing pain over the long term in people with low back pain when combined with standard physical therapy and effective in reducing gastroesophageal reflux symptoms over the short term.

Most studies presented a high risk of bias, had small sample sizes, and therefore they were underpowered to identify statistical differences for evaluated outcomes. It is important to highlight that small sample sizes increase the possibility of type II error, where the likelihood of a study producing a false negative result will be high [32]. The included studies analyzed different visceral manipulations and outcomes. They were extremely diverse in terms of population, type of FTTVS, control groups, outcome measures, the timing of follow-up, and data presentation. FTTVS, like any other manual therapy intervention, varies greatly in technique, pressure, individual treatment times, and an overall number of treatment sessions. Therefore, a meta-analysis was not possible.

Other important clinical aspects of FTTVS that are uncertain are the number of sessions and follow-up period required to generate an effect in the desired outcome. The included studies so far presented interventions with one session [21, 22, 27], two sessions [25], three sessions [23, 24], five sessions [26], 10 sessions [29], 12 sessions [28], 20 sessions [31]. Therefore we believe that it would be of great value that future studies investigate the immediate, short, medium, and long-term effects of FTTVS over different conditions (musculoskeletal, gastrointestinal, cardiac, respiratory, neurological, and urogenital), because it is unclear whether the FTTVS is applied based on the organ or system being treated, i.e., how many sessions should a clinician expect to perform of FTTVS when treating a person with musculoskeletal dysfunction? And for a person with gastrointestinal dysfunction, such as constipation? On a daily basis, clinicians are questioned by colleagues and patients of how many sessions will be required for discharge. Will it be based on clinical symptoms, such as pain in musculoskeletal conditions or an organ function such as in gastroesophageal reflux? Is the discharge based on structure, function, or both? Therefore, clinical questions remain, such as, how many sessions of FTTVS should a clinician perform to treat a person with musculoskeletal dysfunction? And for a person with gastrointestinal dysfunction, such as constipation?

Interestingly, regarding the follow-up period, most studies did not show positive effects of FTTVS, however only two low-risk-of-bias studies demonstrated efficacy for the treatment of patients with low back pain [28] and patients with gastroesophageal reflux [25], the former had a positive long-term effect − 52 weeks after the intervention, the later has a positive short-term effect - one week after the intervention. In general, this raises the question if an intervention that is focused on the improvement of the visceral function itself is an effect faster than for musculoskeletal conditions. All these questions must be answered if we want to practice based on evidence.

One of the major topics in manual therapy is to establish an adequate sham intervention. Eight studies performed sham manual therapy, however, each one performed it differently, demonstrating how this topic is still in debate and needs to be addressed. The sham intervention is defined as an intervention that does not have the same specificity and effect as the intervention technique [33]. Therefore, regarding the specific effect, setup, and condition required for the experimental intervention, then one must exclude these variables to create the sham intervention and apply it in the same place as the experimental intervention. In a recent study by Giandomenico et al. 2022, they suggest describing the following items for both intervention and sham groups: (i) type of touch; (ii) amount of pressure; (iii) type of movement and patient positioning; (iv) areas of contact; (v) time of contact; and (vi) practitioner’s characteristics [33]. In some of the included studies [21, 24–26, 28] was performed the sham technique over the same places as the experimental techniques, but superficially without applying pressure over deep structures. However, they did not describe what were the intervention’s goals, how the operator performed the technique, nor the strategies used by the researcher to avoid the specificity in the sham technique. Stepnik [27] and De Marco [31] performed the sham treatment but in a different place from where they treated the thoracic and pelvic viscera, respectively. In the end, they also did not describe all the items required to justify the differences between the experimental and control interventions. On the other hand, Thomaz, Piche, and Yosri [22, 23, 30] did not perform any type of intervention or sham manual therapy intervention for the control group, except for Yosri [30] who only kept the control group’s participants in the same diet as the experimental group (diet and manual therapy).

As discussed previously, the variety of interventions varied to a great extent, making it difficult to compare the techniques. In general, we could divide the studies between those that performed global techniques (no tissue specificity regarding the outcome and that mixed the visceral techniques with structural and cranial techniques) and specific techniques (applied over the area of the body related to the patient dysfunction and investigated outcome). Attali, Thomaz, Piche, and Tamer [21–23, 29] applied global approaches and had no significant statistical differences when comparing the groups over time. Stepnik, Neto, De Marco, and Lagrange [24, 26, 27, 31] used local approaches, and investigated different conditions but did not have significant statistical differences between the groups over time. However, Yosri, Panagopoulos, and Eguaras [25, 28, 30] showed significant differences between the groups over time. As discussed previously, Panagopoulos [28] and Eguaras [25] were the only two studies with an adequate performance of an RCT study, demonstrating a low risk of bias. This situation makes us wonder if these results are due to the studies’ methodological qualities or because of their specification in treating a condition as both are in the specific group than in the global group. Perhaps this could lead future research to investigate further treatment specificity, going global or specific.

Although a wide variety of conditions are being treated with FTTVS, it is important to have evidence to support these actions. The experimental studies of this review can serve as a starting point for future research, demonstrating some potential conditions that fascial therapy techniques targeting the visceral system can treat effectively.

Finally, future researchers should use the Cochrane risk of bias tool to create a well-designed efficacy study. Furthermore, the CONSORT checklist [34] can aid in the execution of a rigorous randomized controlled clinical trial.

## Conclusion

This systematic review underscores the current lack of strong evidence supporting the efficacy of techniques used in FTTVS. These findings emphasize the need to improve research methodological standards in manual therapies and to conduct more high-quality studies, particularly in the field of visceral osteopathy. It is important to acknowledge that as more research is conducted and the literature base grows, updates to this review will be necessary to provide clinicians with the most up-to-date and reliable information.

## Electronic supplementary material

Below is the link to the electronic supplementary material.

Supplementary Material 1

## Data Availability

The datasets used in the current study are available from the corresponding author upon reasonable request. Request to use such data must be sent to ronaldocruvinel@usp.br.

## References

[1] Gaskin DJ, Richard P (2012). The economic costs of pain in the United States. J Pain.

[2] Johannes CB, Le TK, Zhou X, Johnston JA, Dworkin RH (2010). The prevalence of chronic pain in United States adults: results of an internet-based survey. J Pain.

[3] Rustøen T, Wahl AK, Hanestad BR, Lerdal A, Paul S, Miaskowski C (2004). Prevalence and characteristics of chronic pain in the general norwegian population. Eur J Pain.

[4] Wahl AK, Rustøen T, Rokne B, Lerdal A, Knudsen Ø, Miaskowski C, Moum T (2009). The complexity of the relationship between chronic pain and quality of life: a study of the general norwegian population. Qual Life Res.

[5] Smith MD, Russell A, Hodges PW (2014). The relationship between incontinence, breathing disorders, gastrointestinal symptoms, and back pain in women: a longitudinal cohort study. Clin J Pain.

[6] Aziz Q, Giamberardino MA, Barke A, Korwisi B, Baranowski AP, Wesselmann U, Rief W, Treede RD, Pain IT (2019). f. t. C. o. C. The IASP classification of chronic pain for ICD-11: chronic secondary visceral pain. Pain.

[7] Barral J, Mercier P. *Viscerale Manipulation*; Gent, 1994.

[8] Vleminckx M, Visceral. mobilization. *Carriere B, Markel Feldt C, editors. The pelvic floor. New York: Thieme* 2006, 230–252.

[9] Finet G, Williame C. Treating visceral dysfunction: an Osteopathic Approach to understanding and treating the Abdominal Organs. Stillness Press; 2000.

[10] Fleischmann M (2020). Use of manual therapy applied to the viscera: secondary analysis of a nationally representative sample of australian osteopaths. Int J Osteopath Med.

[11] Organization WH. *Benchmarks for training in traditional / complementary and alternative medicine: benchmarks for training in osteopathy*; 2010.

[12] McSweeney TP, Thomson OP, Johnston R (2012). The immediate effects of sigmoid colon manipulation on pressure pain thresholds in the lumbar spine. J Bodyw Mov Ther.

[13] Silva ACO, Biasotto-Gonzalez DA, Oliveira FHM, Andrade AO, Gomes CAFP, Lanza FC, Amorim CF, Politti F. Effect of Osteopathic Visceral Manipulation on Pain, Cervical Range of Motion, and Upper Trapezius Muscle Activity in Patients with Chronic Nonspecific Neck Pain and Functional Dyspepsia: A Randomized, Double-Blind, Placebo-Controlled Pilot Study. *Evid Based Complement Alternat Med* 2018, *2018*, 4929271. DOI: 10.1155/2018/4929271. 10.1155/2018/4929271 PMC6252226 30534176

[14] Oliva-Pascual-Vaca Á, González-González C, Oliva-Pascual-Vaca J, Piña-Pozo F, Ferragut-Garcías A, Fernández-Domínguez JC, Heredia-Rizo AM. Visceral origin: an underestimated source of Neck Pain. A systematic scoping review. Diagnostics (Basel). 2019;9(4). 10.3390/diagnostics9040186. 10.3390/diagnostics9040186 PMC6963844 31726685

[15] Guillaud A, Darbois N, Monvoisin R, Pinsault N (2018). Reliability of diagnosis and clinical efficacy of visceral osteopathy: a systematic review. BMC Complement Altern Med.

[16] Lo Basso F, Pilzer A, Ferrero G, Fiz F, Fabbro E, Oliva D, Cazzarolli C, Turrina A (2021). Manual treatment for kidney mobility and symptoms in women with nonspecific low back pain and urinary infections. J Osteopath Med.

[17] Moher D, Liberati A, Tetzlaff J, Altman DG, Group* P. Preferred reporting items for systematic reviews and meta-analyses: the PRISMA statement. Ann Intern Med. 2009;151(4):264–9. 10.7326/0003-4819-151-4-200908180-00135 19622511

[18] Higgins JPT, Chandler TJ, Cumpston J, Li M, Page T, Welch MJ VA, editors. Cochrane Handbook for Systematic Reviews of Interventions version 6.3. 2022.

[19] Ouzzani M, Hammady H, Fedorowicz Z, Elmagarmid A (2016). Rayyan—a web and mobile app for systematic reviews. Syst reviews.

[20] Sterne JA, Savović J, Page MJ, Elbers RG, Blencowe NS, Boutron I, Cates CJ, Cheng H-Y, Corbett MS, Eldridge SM. RoB 2: a revised tool for assessing risk of bias in randomised trials. *bmj* 2019, *366*. 10.1136/bmj.l4898 31462531

[21] Attali TV, Bouchoucha M, Benamouzig R (2013). Treatment of refractory irritable bowel syndrome with visceral osteopathy: short-term and long‐term results of a randomized trial. J Dig Dis.

[22] Thomaz SR, Teixeira FA, de Lima ACGB, Cipriano Júnior G, Formiga MF, Cahalin LP (2018). Osteopathic manual therapy in heart failure patients: a randomized clinical trial. J Bodyw Mov Ther.

[23] Piche T, Pishvaie D, Tirouvaziam D, Filippi J, Dainese R, Tonohouhan M, DeGalleani L, Nébot-Vivinus M-H, Payrouse J-L, Hébuterne X (2014). Osteopathy decreases the severity of IBS-like symptoms associated with Crohn’s disease in patients in remission. Eur J Gastroenterol Hepatol.

[24] Lagrange A, Decoux D, Briot N, Hennequin A, Coudert B, Desmoulins I, Bertaut A (2019). Visceral osteopathic manipulative treatment reduces patient reported digestive toxicities induced by adjuvant chemotherapy in breast cancer: a randomized controlled clinical study. Eur J Obstet Gynecol Reprod Biol.

[25] Eguaras N, Rodríguez-López ES, Lopez-Dicastillo O, Franco-Sierra M, Ricard F. Oliva-Pascual-Vaca, Á. Effects of Osteopathic Visceral treatment in patients with gastroesophageal reflux: a Randomized Controlled Trial. J Clin Med. 2019;8(10). 10.3390/jcm8101738. 10.3390/jcm8101738 PMC6832476 31635110

[26] Neto HP, Borges RA. Visceral mobilization and functional constipation in stroke survivors: a randomized, controlled, double-blind, clinical trial. Cureus 2020, *12* (5). 10.7759/cureus.8058 PMC7286593 32537276

[27] Stępnik J, Kędra A, Czaprowski D (2020). Short-term effect of osteopathic manual techniques (OMT) on respiratory function in healthy individuals. PLoS ONE.

[28] Panagopoulos J, Hancock MJ, Ferreira P, Hush J, Petocz P (2015). Does the addition of visceral manipulation alter outcomes for patients with low back pain? A randomized placebo controlled trial. Eur J Pain.

[29] Tamer S, Öz M, Ülger Ö (2017). The effect of visceral osteopathic manual therapy applications on pain, quality of life and function in patients with chronic nonspecific low back pain. J Back Musculoskelet Rehabil.

[30] Yosri MM, Hamada HA, Yousef AM (2022). Effect of visceral manipulation on menstrual complaints in women with polycystic ovarian syndrome. J Osteopath Med.

[31] De Marco M, Arbieto ER, Da Roza TH, Resende AP, Santos GM (2022). Effects of visceral manipulation associated with pelvic floor muscles training in women with urinary incontinence: a randomized controlled trial. Neurourol Urodyn.

[32] Sim J, Wright C. Research in health care: concepts, designs and methods. Nelson Thornes; 2000.

[33] Giandomenico DA, Nuria R, Alessandro A, Matteo G, Mattia I, Marco T, Francesco C (2022). Differences between experimental and placebo arms in manual therapy trials: a methodological review. BMC Med Res Methodol.

[34] Schulz KF, Altman DG, Moher D (2010). CONSORT 2010 statement: updated guidelines for reporting parallel group randomised trials. J Pharmacol pharmacotherapeutics.

