# Supplementary material for: Effectiveness of visceral fascial therapy targeting visceral dysfunctions outcome: systematic review of randomized controlled trials
Source: BMC Complement Med Ther. 2023 Jul 31;23:274. doi: 10.1186/s12906-023-04099-1 (PMC10391879; doi:10.1186/s12906-023-04099-1)
Supplement: Supplementary file 1 — Supplementary Material 1 [file 12906_2023_4099_MOESM1_ESM.docx]

**Additional File 1: Summary of Search Strategy Across all Databases**

The literate search was conducted up to August 2022. A systematic review of the literature was performed following the Preferred Reporting Items for Systematic Reviews and Meta-Analyses (PRISMA). PubMed (National Library of Medicine), PEDro (Physiotherapy Evidence Database), SciELO, BVS Bireme, Cochrane , CENTRAL, Osteopathic Research Web, Journal of Osteopathic Medicine (JOM) website, and OSTMED.D databases using the following keywords:

| **“**Manipulation, Osteopathic” [MeSH Tems]  **OR “**Osteopathic Manipulative Treatment” [MeSH Tems]  **OR** Osteopathic Manipulative Treatments [MeSH Tems]  **OR** Treatment, Osteopathic Manipulative [MeSH Tems]  **OR** Treatments, Osteopathic Manipulative [MeSH Tems]  **OR** Osteopathic Manipulation [MeSH Tems]  **OR** Visceral Manipulation [Text Word]  **OR** Visceral Osteopathic Manipulation [Text Word]  **OR** Visceral Manual Therapy [Text Word]  **OR** Visceral Osteopathic Manual Therapy [Text Word]  **OR** Visceral Osteopathy [Text Word]  **OR** Visceral Osteopathic Manipulative Treatment [Text Word]  **OR** Visceral Osteopathic treatment [Text Word] | **AND** | “Clinical Trials, Randomized” [MeSH Tems]  **OR** “Trials, Randomized Clinical” [MeSH Tems]  **OR** “Controlled Clinical Trials, Randomized” [MeSH Tems]  **OR** RCT [Text Word] |
| --- | --- | --- |
